# A Field Test of Major Value Frameworks in Chemotherapy of Nasopharyngeal Carcinoma—To Know, Then to Measure

**DOI:** 10.3389/fonc.2020.01076

**Published:** 2020-08-12

**Authors:** Yuan Zhang, Xu Liu, Ying-Qin Li, Ling-Long Tang, Lei Chen, Jun Ma

**Affiliations:** ^1^State Key Laboratory of Oncology in South China, Guangdong Key Laboratory of Nasopharyngeal Carcinoma Diagnosis and Therapy, Department of Radiation Oncology, Collaborative Innovation Center of Cancer Medicine, Sun Yat-sen University Cancer Center, Guangzhou, China; ^2^Department of Radiation Oncology, University of Texas M.D. Anderson Cancer Center, Houston, TX, United States

**Keywords:** value framework, European Society for Medical Oncology, Magnitude of Clinical Benefit Scale, American Society of Clinical Oncology, drug therapy, nasopharyngeal neoplasms

## Abstract

**Background:** The European Society for Medical Oncology (ESMO) and the American Society of Clinical Oncology (ASCO) have independently developed their own frameworks to assess the benefits of different cancer treatment options, which have significant implications in health science and policy. We aimed to compare these frameworks in nasopharyngeal carcinoma.

**Methods:** We identified all randomized controlled trials of systemic chemotherapies for nasopharyngeal carcinoma until April 5th, 2020. Trials were eligible if significant differences favoring the experimental group in a prespecified primary or secondary outcome were reported. Two assessors independently scored the trials and the final scores were determined by consensus.

**Results:** Fifteen trials were included in the analysis. Five different toxicity grading criteria were applied to the 15 trials. Ten (66.7%) trials did not report grade 1–2 toxicities and eight (53.3%) did not report late toxicities. The number of acute toxicities reported was strikingly different (17 vs. 8) in two trials using the same regimen. All trials met the ESMO criteria for a high level of benefit. However, significant variations in ASCO scores between trials were observed (mean [standard deviation]: 38.9 [20.0]).

**Conclusions:** The underreporting and inconsistent reporting of toxicities would significantly impair the assessment of value using any framework. Moreover, there is a concern that the ASCO framework generated highly inconsistent scoring for treatments that met the ESMO criteria for a high level of benefit. The anomalies identified in the frameworks function would be helpful in their future improvement.

## Introduction

The goal of any cancer therapy is to help patients live longer, or live better, or both. In the clinic, oncologists, and patients need to discuss the balance of benefit and toxicity associated with different treatment options, to make the best decision for each patient. The European Society for Medical Oncology (ESMO) and the American Society of Clinical Oncology (ASCO) have proposed and updated frameworks to assess the value of cancer treatment options (1, 2).

Nasopharyngeal carcinoma (NPC) is prevalent in Southern China, Southeast Asia, North Africa, the Middle East, and Alaska (3). Radiotherapy (RT) is the primary treatment for non-metastatic NPC. Multiple randomized controlled trials (RCTs) have shown that combining chemotherapy with RT improves outcome in loco-regionally advanced NPC. However, different sequences (induction, concurrent, adjuvant, and their combinations) and regimens of chemotherapy were used in these RCTs and controversy remains over which treatment option is optimal (4). In recurrent or metastatic NPC, chemotherapy is the mainstay of treatment and various regimens have been used in the clinic.

Recently, researchers have used the ESMO and ASCO frameworks to assess systemic therapies for cancers (5–8). However, to the best of our knowledge, no study has tested these frameworks in NPC. We applied the updated ESMO and ASCO value frameworks to RCTs investigating systemic chemotherapies in NPC.

## Materials and Methods

### Literature Search

This systematic analysis aimed to include all relevant published trials on systemic chemotherapies in NPC. The following electronic databases were searched to identify potentially eligible trials: PubMed, Web of Science, and the Central Registry of Controlled Trials of the Cochrane Library (CENTRAL). The search was supplemented by a manual search of the reference lists of primary studies, review articles, meta-analyses, and relevant books. To search PubMed and Web of Science, we adopted a search algorithm used in the latest individual patient data meta-analysis of chemotherapy in NPC (4). For CENTRAL, we used the Medical Subject Heading “nasopharyngeal neoplasms” to search for studies. The language and time were not limited in the search, which was performed on April 5th, 2020.

The search algorithms were as follows:

PubMed:
  ((nasopharyngeal neoplasms/drug therapy [MAJR] OR nasopharyngeal neoplasms/radiotherapy [MAJR]) AND (clinical trial [Publication Type] AND (random^*^ OR (Phase III)Fields: Title Word))) OR ((nasopharyngeal neoplasms/drug therapy [MAJR] OR nasopharyngeal neoplasms/radiotherapy [MAJR]) AND (clinical trial, phase III [Publication Type] OR randomized controlled trial [Publication Type]))
Web of Science:
  TS = (nasopharyn^*^ OR cavum) AND TS = (chemotherapy OR chemoradiation OR chemoradiotherapy OR radiochemotherapy OR radio-chemotherapy OR pharmacotherapy) AND TS = (cancer^*^ OR carcinoma^*^ OR adenocarcinoma^*^ OR malignan^*^ OR tumor^*^ OR tumor^*^ OR neoplasm) AND TS = (random^*^) AND TS = (trial^*^) NOT TS = (retrospective^*^)
Refined by: DOCUMENT TYPES: (CLINICAL TRIAL)
Timespan: All years. Databases: WOS.
CENTRAL:
#1 = MeSH descriptor: [Nasopharyngeal Neoplasms] explode all trees
#2 = random^*^
#3 = #1 and #2

### Study Selection

The following criteria were applied to the selection of RCTs:

1. RCTs reporting significant differences favoring the experimental group in a prespecified primary or secondary outcome. Trials with “negative” results were excluded, as they were not assessable according to the frameworks. This is in accordance to the ESMO-Magnitude of Clinical Benefit Scale (MCBS) version 1.1 stating that only “adequately powered studies showing statistically significant improvement in the primary outcomes or secondary outcomes” should be scored (2).
2. At least 50% of trial participants had NPC;
3. At least 30 patients had been included in each arm;
4. Trials using split-course RT were excluded.

Two authors (YZ and XL) independently performed the literature search and study selection. Any inconsistencies were discussed until consensus was reached.

### Frameworks

The updated ASCO–Value Framework (ASCO-VF) and ESMO-MCBS both quantify gains in overall survival (OS) or its surrogates (e.g., disease-free survival [DFS]) (1, 2). In ASCO-VF, the hazard ratio (HR) is subtracted from one and the result is multiplied by 100 to derive a Clinical Benefit Score; in ESMO-MCBS, HRs, and/or survival gains are linked to a particular grade in a pre-specified manner. For example, in the curative setting, a >5% improvement of OS at ≥3 years follow-up translates to a grade of A. Both scales use different forms for treatment in curative and palliative setting.

Toxicity and quality-of-life (QoL) data are used to adjust the scores or grades in both frameworks. For ASCO-VF, different points are assigned to every “clinically meaningful toxicity” based on its frequency and severity (e.g., 2.0 points for every grade 3 or 4 toxicity with a frequency ≥5%). The percentage difference in the sum of toxicity points between the two regimens is then multiplied by −20 to obtain a Toxicity Score. If the test regimen is more toxic than the comparator, the toxicity score is negative and vice versa. In the ESMO-MCBS, some prespecified severe toxicities are explicitly outlined and grades reduced by 1 level if toxic effects meet any of these prespecifications (e.g., a statistically significant increase of toxic death rate >2%).

Both frameworks award bonus for a “tail of the survival curve effect.” The ASCO-VF award 16–20 bonus points if there is a 50% or greater improvement in the proportion of patients alive with the test regimen at the time point on the survival curve that is 2 × the median survival of the comparator regimen. The ESMO-MCBS requires an upgrade of 1 level if there is a long-term plateau in the survival curve. Final ASCO-VF scores, termed Net Health Benefit, are the sum of Clinical Benefit Score, Toxicity Score and any bonus points (possible range −20 to more than 120 with bonus point allocation); ESMO-MCBS grades are ranked C, B, or A (for the curative setting), and 1, 2, 3, 4, or 5 (for the palliative setting). ESMO-MCBS defines “substantial clinical benefit” as a grade of 4, 5, B, or A whereas ASCO-VF includes no explicit definition.

### Data Abstraction, Scoring, and Statistical Analysis

Firstly, two assessors (YZ, XL) independently scored the trials according to both frameworks. Secondly, the two assessors discussed the results and determined the final scores by consensus. Bias in trials was evaluated by one assessor (XL) using the Cochrane risk of bias assessment tool (9). Data were collected in an Excel file designed for this study. Descriptive statistics were used to summarize the scoring.

## Results

**Figure 1:**
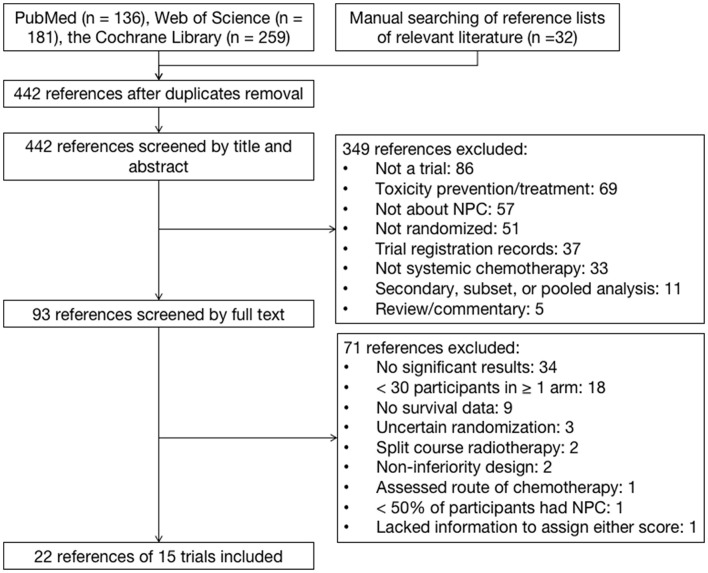
Identification of trials on systemic chemotherapies in patients with nasopharyngeal carcinoma.

The electronic and manual search identified 195 references after the removal of duplicates. After screening, 22 references for 15 trials were eligible (Figure 1). Only one study was excluded because of insufficient information to assign a score for either framework. The median sample size of the 13 included trials was 284. Eleven trials investigated chemotherapy in the curative treatment of non-metastatic NPC, including four trials comparing concurrent chemoradiation (CCRT) plus adjuvant chemotherapy (AC) vs. RT alone, four trials comparing CCRT vs. RT alone, and five trials comparing induction chemotherapy (IC) plus CCRT vs. CCRT (Table 1). Two trials investigated palliative treatment of recurrent or metastatic NPC: one compared cisplatin and gemcitabine vs. cisplatin and fluorouracil and the other compared cisplatin and fluorouracil every 2 weeks vs. every 4 weeks (Table 2).

**Table 1:** Summary of trials in the curative treatment of non-metastatic, newly diagnosed nasopharyngeal carcinoma.

**Study**	**No. of Pts**	**Stage**	**RT Technique**	**RT Dose (Gy)**	**Chemotherapy Regimen**		
					**Induction**	**Concurrent**	**Adjuvant**
**CCRT+AC vs. RT**							
INT-0099 (10)	193	III–IV	2D	70	None	Cisplatin 100 D1 Q3W*3	Cisplatin 80 D1; 5FU 1,000 D1–4 Q4w*3
SQNP01 (11)	221	III–IV	2D	70	None	Cisplatin 25 D1–4 Q3W*3	Cisplatin 20 D1–4; 5FU 1,000 D1–4 Q4w*3
NPC-9901 (12–14)	348	Any T, N2–3	Mixed 2D and conformal	≥66	None	Cisplatin 100 D1 Q3W*3	Cisplatin 80 D1; 5FU 1,000 D1–4 Q4W*3
SYSUCC-02 (15, 16)	316	III–IV	2D	>66	None	Cisplatin 40 D1 QW during RT	Cisplatin 80 D1; 5FU 800 D1–5 Q4W*3
**CCRT vs. RT**							
TVGH-93 (17)	284	III–IV	2D	70–74	None	Cisplatin 20 D1–4; 5FU 400 D1–4 Q4W*2	None
PWHQEH-94 (18, 19)	350	II–IV	2D	66	None	Cisplatin 40 QW*8	None
SYSUCC-01 (20, 21)	115	III–IV	2D	70–74	None	Oxaliplatin 70 D1 QW*6	None
SYSUCC-03 (22)	230	II–III	2D	68–70	None	Cisplatin 30 QW during RT	None
**IC+CCRT vs. CCRT**							
NPC-008 (23)	65	III–IVB	Mixed 2D and IMRT	66	Docetaxel 75 D1; Cisplatin 75 D1 Q3w*2	Cisplatin 40 QW during RT	None
GORTEC 2006-02 (24)	83	T2b-4 and/or N1–N3	Mixed IMRT and non-IMRT (not specified)	70	Docetaxel 75 D1; Cisplatin 75 D1; 5FU 750 D1–5 Q3w*3	Cisplatin 40 QW during RT	None
SYSUCC-PF (25, 26)	476	III-IVB (excluding T3N0–1)	Mixed 2D and IMRT		Cisplatin 80 D1; 5FU 800 D1–5 Q3W*2	Cisplatin 80 D1 Q3W*3	None
SYSUCC-TPF (27, 28)	480	III-IVB (excluding T3–4N0)	IMRT	70	Docetaxel 60 D1; Cisplatin 60 D1; 5FU 600 D1–5 Q3w*3	Cisplatin 100 D1 Q3W*3	None
SYSUCC-GP IC (29)	480	III-IVB (excluding T3–4N0)	IMRT	70	Gemcitabine 1g D1,8; Cisplatin 80mg D1 Q3w*3	Cisplatin 100 D1 Q3W*3	None

*AC, adjuvant chemotherapy; CCRT, concurrent chemoradiotherapy; IC, induction chemotherapy; IMRT, intensity-modulated radiation therapy; INT-0099, Southwest Oncology Group-coordinated Intergroup trial; No., number; NPC, nasopharyngeal carcinoma; Pts, patients; PWHQEH, Prince of Wales Hospital, Queen Elizabeth Hospital; RT, radiotherapy; SQNP, Singapore Naso-Pharynx; SYSUCC, Sun Yat-sen University Cancer Center; 2D, 2-dimension conventional radiotherapy; TVGH, Taichung Veterans General Hospital, Taiwan*.

**Table 2:** Summary of trials in the treatment of recurrent or metastatic nasopharyngeal carcinoma.

**Study**	**No. of patients**	**Eligible patients**	**Experimental arm**	**Control arm**
SYSUCC-GP (30)	362	Recurrent or metastatic	Cisplatin + gemcitabine	Cisplatin + fluorouracil
Guangxi-10 (31)	103	Metastatic	Cisplatin + fluorouracil, every 2 weeks^a^	Cisplatin + fluorouracil, every 4 weeks^a^

*GP, cisplatin and gemcitabine; SYSUCC, Sun Yat-sen University Cancer Center*.

a *After chemotherapy, residual lesions were treated with additional radiotherapy*.

We found significant variation in the reporting of toxicities. Among the 13 trials, five different toxicity grading criteria were used, including criteria developed by the Southwest Oncology Group, the World Health Organization, and the Radiation Therapy Oncology Group, and the National Cancer Institute Common Terminology Criteria for Adverse Events. Ten (66.7%) studies did not report grade 1–2 toxicities and eight (53.3%) did not report late toxicities. The number of acute toxicities reported was strikingly different (17 vs. 8) in two trials using the same regimen (10, 11). Moreover, no trial reported QoL data.

### Scoring With the ESMO-MCBS and ASCO-VF

All 15 trials were assessable with the ESMO-MCBS. Among 13 trials in the curative setting, 12 (92.3%) trials were graded at the highest ESMO grade of A and one trial was grade B. Both trials in the palliative setting were graded at ESMO grade 4. Thus, all trials met the ESMO threshold for substantial benefit.

**Figure 2:**
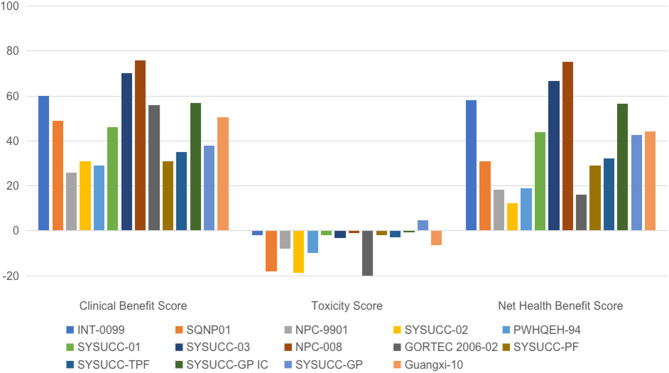
Scoring of trials evaluating chemotherapy in nasopharyngeal carcinoma using the ASCO value framework. ASCO, American Society of Clinical Oncology; GP, cisplatin, and gemcitabine; INT-0099, Southwest Oncology Group-coordinated Intergroup trial; NPC, nasopharyngeal carcinoma; PF, cisplatin, and fluorouracil; PWHQEH, Prince of Wales Hospital, Queen Elizabeth Hospital; SQNP, Singapore Naso-Pharynx; SYSUCC, Sun Yat-sen University Cancer Center, China; TPF, docetaxel, cisplatin, and fluorouracil.

Fourteen of 15 trials were assessable using the ASCO-VF. One trial comparing CCRT vs. RT did not provide HR for survival, meaning it could not be evaluated using the ASCO-VF (17). Another trial comparing IC plus CCRT vs. CCRT reported a statistically significant improvement in the primary endpoint of DFS (HR = 0.44; 95% CI: 0.20–0.97, *p* = 0.042). However, there was no significant difference in OS (HR = 0.40; 95% CI: 0.15–1.04, *p* = 0.059) with a median follow-up of 43.1 months. Because the OS data was not mature, the trial was evaluated on the basis of DFS results after discussion between the two assessors. As shown in Figure 2, significant variations in ASCO Clinical Benefit Score (mean: 46.8; standard deviation [SD]: 15.8), Toxicity Score (mean: 7.8; SD: 11.3), and Net Health Benefit Score (mean: 38.9; SD: 20.0) between trials were noticed.

## Discussion

To the best of our knowledge, this study is the first to test the ESMO and ASCO frameworks in trials evaluating chemotherapy in NPC. We found significant variation in the reporting of toxicities, including different grading criteria and deficiencies in the reporting of grade 1–2 and long-term toxicities. These results are consistent with previous evidence suggesting that the reporting of toxicity data from RCTs needs improvement (32). The underreporting and inconsistent reporting of toxicities would significantly impair the assessment of value using any framework in any possible settings, not only in NPC. Compliance with established guidance on toxicity reporting and sharing of clinical trial data may help mitigate this problem (33, 34). Moreover, subjective toxicities are at high risk of underreporting by physicians, even when prospectively collected within randomized trials (35). This strongly supports the need for incorporation of patient-reported outcomes and QoL data into toxicity reporting in clinical trials (36).

Our two assessors had a perfect agreement in the ESMO-MCBS analysis except in the assessment of one trial in the palliative setting (30). The ESMO-MCBS requires upgrading one level if the new treatment is associated with “statistically significantly less grade 3–4 toxicities impacting on daily well-being” compared with the standard therapy in the non-curative setting. In the trial comparing cisplatin and gemcitabine vs. cisplatin and fluorouracil in recurrent or metastatic NPC (SYSUCC-GP), the overall incidences of grade 3–4 toxicities were not significantly different between the two arms (43.3 vs. 35.8%, *p* = 0.18), while the experimental arm had significantly fewer grade 3–4 mucosal inflammation (0 vs. 14.5%, *p* < 0.001) (30). Our two assessors differed on whether this met the criteria for upgrade. After discussion, they decided that no upgrade should be done. More detailed guidance on this criterion might help avoid discrepancy in the future.

For the ASCO framework, however, wide variation in the initial independent analysis occurred between the two assessors, mainly due to the different interpretation of “clinically meaningful toxicity.” The ASCO-VF defined “clinically meaningful toxicities” as toxicities other than laboratory abnormality only, which might be ambiguous and prone to different interpretations. For example, grade 1–2 hyponatremia may be symptomless while grade 3–4 hyponatremia might cause symptoms like fatigue. A clearer definition would facilitate more consistent scoring, which was also suggested by de Hosson et al. (6).

Our results demonstrated good applicability of both frameworks. Trials included in this study achieved highly consistent grades using the ESMO-MCBS. The ASCO-VF, however, gave very inconsistent and disparate scoring. For example, in the curative setting, all except one trial met the ESMO criteria for the highest level of benefit (grade A), while significant variations were found in the ASCO-VF scoring of Clinical Benefit, Toxicity as well as the final Net Health Benefit.

An important difference between these two frameworks is that the ESMO-MCBS places increasing weights on the toxicity profile as the treatment effects moves from curative to increasing palliative settings, while the calculation of toxicity score in the ASCO-VF is the same regardless of curative or palliative setting. For example, in the curative setting, for a new treatment regimen that improved the OS by >5%, the ESMO-MCBS would score a grade of A regardless of toxicity, while the ASCO-VF would take toxicity into consideration. In theory, the ASCO approach might be more reasonable. However, this is also part of the reason why the ASCO-VF score has significant variations. Conversely, unlike the ESMO-MCBS, the ASCO-VF didn't mention grade 5 toxicity (treatment-related death), which we believe is of vital importance in assessing toxicities.

For ASCO-VF, each toxicity is assigned a score between 0.5 and 2.0, based on grade and frequency. However, these points are arbitrary, not intuitive, and this may have obscured the actual differences in toxicity. For example, in the PWHQEH-94 trial comparing CCRT vs. RT alone, grade 3–4 stomatitis was observed in 48.9 and 35.8% of patients in the CCRT and RT-only groups, respectively, with a significant difference of 13.1% (*p* = 0.002) (18). However, when grading using the ASCO criteria, both groups scored two points, despite the apparent clinically relevant difference. In the original ASCO framework, the HR for survival was also assigned a score of 1–5 on the basis of the magnitude of difference (e.g., 5 for an HR <0.2). While in the revised framework, a continuous scoring system is used to avoid arbitrary cut-offs (1). In the same vein, a continuous scoring system for toxicity might more accurately reflect the absolute difference in toxicity, as shown in Table 3. Such calculations could be easily performed once the framework is converted to a software application, as planned by ASCO (1).

**Table 3:** Comparison of toxicity assessment using the current ASCO value framework and a proposed continuous system^a^.

	**Radiotherapy alone arm (*****n*** **=** **176)**		**Concurrent chemoradiotherapy arm (*****n*** **=** **174)**	
**Stomatitis**	**Grade 3**	**Grade 4**	**Grade 3**	**Grade 4**
Incidence (%)	34.7	1.1	44.3	4.6
**Current ASCO framework**				
Toxicity points	2		2	
Percentage difference	0			
Toxicity score^b^	0			
**A proposed continuous system**				
Toxicity points	3 × 0.347 = 1.041	4 × 0.011 = 0.044	3 × 0.443 = 1.329	3 × 0.046 = 0.138
Sum	1.085		1.467	
Percentage difference	1.467/1.085 – 1 = 0.352			
Toxicity score^b^	0.352 × 20 = 7.04			

*ASCO, American Society of Clinical Oncology*.

a *Based on data in the PWHQEH-94 trial comparing concurrent chemoradiotherapy vs. radiotherapy alone (18)*.

b *toxicity score = 20 × the percentage difference in toxicity points between the two regimens, according to the ASCO value framework*.

The study had some limitations. Firstly, only trials reporting significant results favoring the experimental arm were assessable using the frameworks. However, our study was a field test of ESMO and ASCO frameworks in systemic chemotherapy of NPC and not aimed at determining the value of different treatment options. A balanced value assessment requires the consideration of all relevant studies, whether they report significant findings or not, which was beyond the scope of this study. Secondly, our research was limited to RCTs investigating systemic chemotherapy in NPC; the applicability of value frameworks in other treatments or other diseases might be different. Nevertheless, there is a strong probability that similar situations apply to other settings. Thirdly, no trials included in the current study reported QoL data. It was not clear how such data will impact value assessments. Finally, only 6 of 13 trials in the curative setting used intensity-modulated radiotherapy, which has become the standard of care in NPC (37).

In conclusion, significant variations regarding toxicity reporting were found in trials evaluating chemotherapy in NPC. Both frameworks could be applied to the systemic chemotherapy of NPC. However, there is concern that the ASCO-VF generated highly inconsistent scoring for treatments that met the ESMO criteria for high level of benefit. The successful future application of value frameworks requires consistent reporting of toxicities as well as iterative refining and intergroup alignment of different frameworks.

## Data Availability Statement

The raw data supporting the conclusions of this article will be made available by the authors, without undue reservation.

## Author Contributions

Study design: YZ and JM. Data collection: YZ, XL, and Y-QL. Revision of the manuscript, data analysis, and interpretation: All authors. Writing of the manuscript: YZ. Statistical analysis: YZ, XL, and Y-QL.

## Conflict of Interest

The authors declare that the research was conducted in the absence of any commercial or financial relationships that could be construed as a potential conflict of interest.
